# Transcriptome analysis reveals *in vitro* cultured *Withania somnifera* leaf and root tissues as a promising source for targeted withanolide biosynthesis

**DOI:** 10.1186/s12864-015-1214-0

**Published:** 2015-01-22

**Authors:** Kalaiselvi Senthil, Murukarthick Jayakodi, Pankajavalli Thirugnanasambantham, Sang Choon Lee, Pradeepa Duraisamy, Preethi M Purushotham, Kalaiselvi Rajasekaran, Shobana Nancy Charles, Irene Mariam Roy, Arul Kumar Nagappan, Gon Sup Kim, Yun Sun Lee, Senthil Natesan, Tae-Sun Min, Tae Jin Yang

**Affiliations:** Department of Biochemistry, Biotechnology and Bioinformatics, Avinashilingam Institute for Home Science and Higher Education for Women, Coimbatore, 641043 India; Department of Plant Science, Plant Genomics and Breeding Institute, Research Institute for Agriculture and Life Sciences, College of Agriculture and Life Sciences, Seoul National University, Seoul, 151-921 Republic of Korea; Lab of Biochemistry, School of Veterinary Medicine, Gyeongsang National University, Gyeongsangnam-do, Republic of Korea; Genomics and Proteomics Laboratory, Centre for Plant Molecular Biology & Biotechnology, Tamil Nadu Agricultural University, Coimbatore, 641003 Tamil Nadu India; National Research Foundation, Seoul, Republic of Korea

**Keywords:** Withanolides, Transcriptome, Illumina platform, Hiseq, *in vitro* adventitious roots

## Abstract

**Background:**

The production of metabolites via *in vitro* culture is promoted by the availability of fully defined metabolic pathways. Withanolides, the major bioactive phytochemicals of *Withania somnifera,* have been well studied for their pharmacological activities. However, only a few attempts have been made to identify key candidate genes involved in withanolide biosynthesis. Understanding the steps involved in withanolide biosynthesis is essential for metabolic engineering of this plant to increase withanolide production.

**Results:**

Transcriptome sequencing was performed on *in vitro* adventitious root and leaf tissues using the Illumina platform. We obtained a total of 177,156 assembled transcripts with an average unigene length of 1,033 bp. About 13% of the transcripts were unique to *in vitro* adventitious roots but no unique transcripts were observed in *in vitro*-grown leaves. A putative withanolide biosynthetic pathway was deduced by mapping the assembled transcripts to the KEGG database, and the expression of candidate withanolide biosynthesis genes -were validated by qRT PCR. The accumulation pattern of withaferin A and withanolide A varied according to the type of tissue and the culture period. Further, we demonstrated that *in vitro* leaf extracts exhibit anticancer activity against human gastric adenocarcinoma cell lines at sub G1 phase.

**Conclusions:**

We report here a validated large-scale transcriptome data set and the potential biological activity of *in vitro* cultures of *W. somnifera*. This study provides important information to enhance tissue-specific expression and accumulation of secondary metabolites, paving the way for industrialization of *in vitro* cultures of *W. somnifera*.

**Electronic supplementary material:**

The online version of this article (doi:10.1186/s12864-015-1214-0) contains supplementary material, which is available to authorized users.

## Background

*Withania somnifera* (L.) Dunal (Family, Solanaceae) commonly known as Ashwgandha or winter cherry is one of the top medicinal herbs used in ayurveda – the holistic system of Indian medicine. Most of its therapeutic properties are comparable to those of ginseng, and hence *W. somnifera* is also known as Indian ginseng. *W. somnifera* extract in various forms has been used as an adaptogen, aphrodisiac, liver tonic, anti-inflammatory agent, hepatoprotectant, astringent, immune booster [1], immunomodulator [2] and more recently to treat Alzheimer’s disease [3], neurodegenerative disorders and stress [4] and also as an adjunct to chemotherapy and/or radiation therapy [5,6]. Wide variations in the type and content of secondary metabolites have been reported within chemotypes and parts of the *W. somnifera* plant [7,8]. The principle bioactive components, a group of secondary metabolites collectively called withanolides, are C-28 steroidal lactones [9]. Withanolides are biosynthesised through the isoprenoid pathway, probably via both the mevalonate and non-mevalonate pathways [8] wherein 24-methylene cholesterol is the first branching point towards the biosynthesis of different withanolides through a series of desaturation, hydroxylation, epoxidation, cyclization, chain elongation, and glycosylation steps [10,11].

*W. somnifera* has been identified by the National Medicinal Plant Board of India as one of the thirty-two priority medicinal plants that are in great demand in the domestic and international markets [12]. According to one estimate, the demand for withanolide production from dried plant material is 12,120 tonnes [13], whereas the annual production is 5,905 tonnes [14]. To meet the growing demand of the herbal industry, *in vitro* cultures could be an alternative to field-grown plants for consistent production of secondary metabolites within a short period [15]. Several reports indicate the potential of manipulating *in vitro* tissues to produce bioactive terpenoids, phenolics, saponins and anthocyanins [16-18], but any attempt to engineer efficient production of secondary metabolites requires understanding of their biosynthetic pathway(s), and our present knowledge of withanolide biosynthesis is limited to only a few genes involved in the pathway [10,19,20].

Next-generation sequencing (NGS) technology for transcriptomes (RNA-seq) provides a new approach for both obtaining gene sequences and quantifying transcriptomes of any organism. In recent years, RNA-seq has been a powerful method for identifying genes involved in important secondary metabolite pathways such as biosynthesis of ginsenosides in *Panax ginseng* [21-24], carotenoids in *Momordica cochinchinensis* [25], flavonoids, theanine and caffeine in tea (*Camellia sinensis*) [26], flavonoids in safflower (*Carthamus tinctorius L.*) [27], capsaicinoid in chili pepper (*Capsicum frutescens*) [28] and flavor components in radish (*Raphanus sativus L.*) [29]. Initial efforts have been made to generate expressed sequence tags (ESTs) from *in vitro* tissues of *W. somnifera* [30]. In addition, leaf and root transcriptomes of field-grown tissues have been analysed to identify genes involved in withanolide biosynthesis [20], and very recently, analysis of the expression of pathogenesis-related genes has been carried out in leaf transcriptomes of *W. somnifera* [31]. However, no large-scale transcriptome information validated by expression profiling is available for *in vitro* tissues of *W. somnifera*.

Here, we used RNA-seq for large-scale transcriptome profiling and generated a comprehensive transcriptome for *W. somnifera* by assembling the transcriptomes of *in vitro* adventitious root and leaf tissues from the millions of short sequence reads generated by Illumina. Annotations including functional annotation, Gene Ontology (GO) and Kyoto Encyclopedia of Genes and Genomes (KEGG) pathways - are also reported. In addition, we showed that *in vitro* tissue extracts have antioxidant activity and cytotoxicity against human adenocarcinoma gastric cancer (AGS) cell lines. Furthermore, we have developed a web database in which to access the *W. somnifera* transcriptome. Overall, this work represents the first large-scale transcriptome profiling of *in vitro* tissues for *W. somnifera* and provides comparative expression profiling of pathway genes involved in withanolide biosynthesis and their potential biological activity.

## Results

### Induction and maintenance of *in vitro* cultures

**Figure 1:**
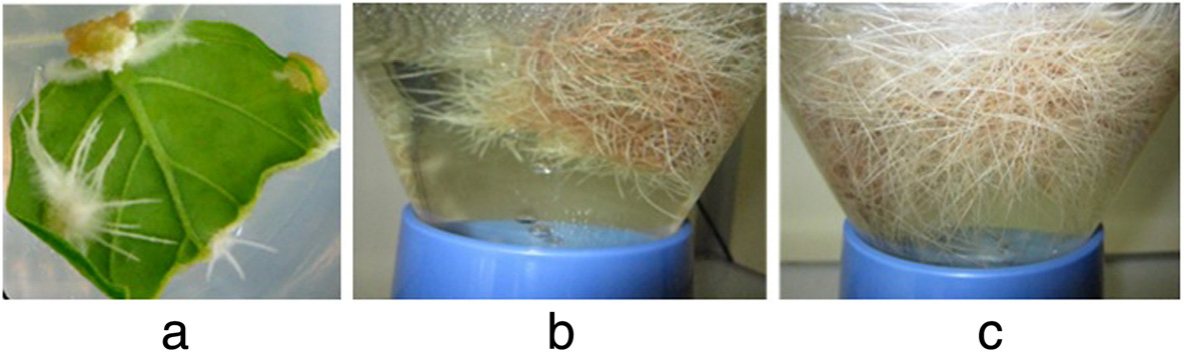
**Adventitious root induction and proliferation in bioreactor. a** -Induction of adventitious roots from the leaf explants of *W. somnifera*, **b** Propagation stage after 30 days of culture, **c** Mass production of root after 45 days in bioreactor.

Direct adventitious roots were induced successfully from leaf explants of *W. somnifera*. Further induced roots were maintained in MS liquid medium in a bubble column bioreactor (Figure 1). At 45 days, adventitious roots showed maximal growth with increased branching. The roots were healthy and creamy white in colour. By 60 days, the roots turned brown and brittle. Leaf tissues from multiple shoots maintained *in vitro* were used for isolation of total RNA.

### Illumina sequencing and *de novo* assembly

To establish a comprehensive overview of the *W. somnifera* transcriptome, we prepared RNA-seq libraries from total RNA isolated from 60-day-old *in vitro* cultures of adventitious root and leaf tissues and subjected them to paired-end (PE) sequencing with the Illumina platform. We obtained 135,186,223 and 113,849,837 cleaned high-quality reads from the *in vitro* adventitious root and leaf tissues, respectively (Table 1). We combined both sets of reads and assembled them using Trinity [32] to generate a unique transcript library. After read depth analysis, we obtained a total of 177,156 transcripts (including isoforms) with an average length of 1,033 bp (Table 1) that we used for subsequent analyses and also prepared a set of 105,662 non-redundant (Nr) transcripts by selecting only the longest sequence among isoforms.

**Table 1:** **Summary of** ***W. somnifera*** **transcriptome sequencing, assembly and annotation**

**Tissues**	**No. of raw reads**	**No. of trimmed reads**	**No. of assembled transcripts**	**Nr set (without isoforms)**	**Average transcript length (bp)**	**Longest transcript length (bp)**
*in vitro* root	139,562,334	135,186,223	177,156	105,662	1,033	10,802
*in vitro* leaf	117,948,186	113,849,837				

### Differentially expressed transcripts between *in vitro*-grown roots and leaves

**Figure 2:**
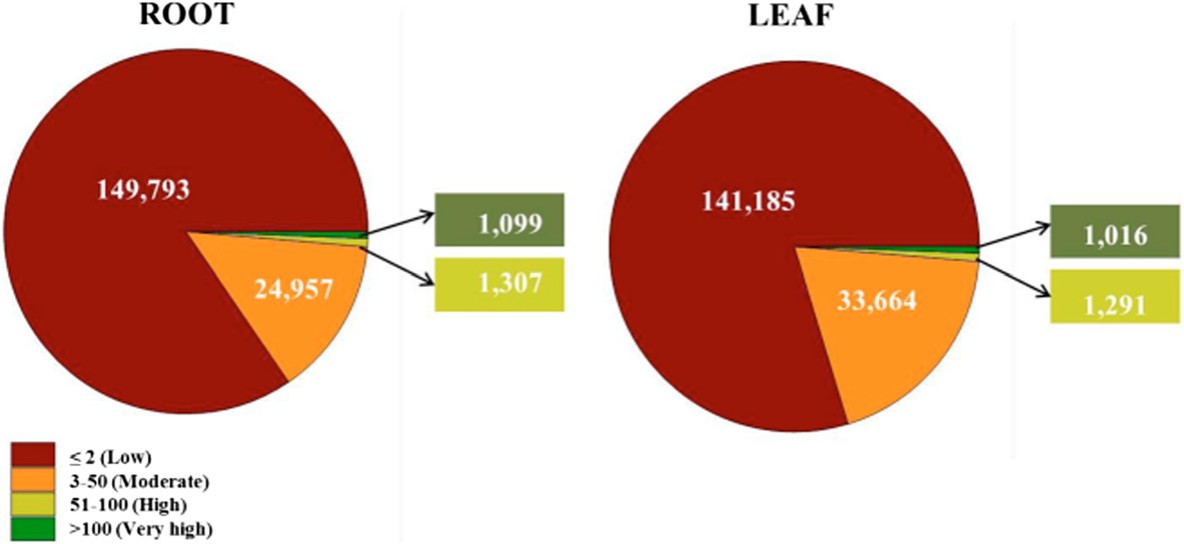
**Expression profiling of** ***W. somnifera*** **.** Root and leaf transcripts were classified as having low, moderate, high, or very high expression based on FPKM values.

High-quality reads from *in vitro* adventitious root and leaf samples were separately mapped to the assembled transcripts and the number of reads mapped was normalized by the FPKM method using RSEM [33]. We classified gene expression into four categories (low, moderate, high and very high), based on the FPKM values for each transcript in the respective tissue samples (Figure 2). The largest fraction of transcripts showed low expression (FPKM 0–2) in both tissue samples. A small fraction (14%-19%) was expressed moderately in both tissues and very few (1%-2%) had high expression (Figure 2). In addition, we identified transcripts expressed specifically in each tissue sample using tissue-by-tissue comparison between root and leaf. For this, we normalized the number of reads mapped to each transcript per million (TPM) mapped for root and leaf tissues and considered transcripts to be expressed specifically in a single tissue (root or leaf) if they showed at least 3 TPM in the tissue of interest and zero in the other. Among the total 177,156 transcripts analysed in this study, a large number (152,839) were present in both tissues and a total of 24,317 were preferentially expressed in root. By contrast, we did not find any transcripts expressed exclusively in leaf. To identify differentially-expressed transcripts, the edgeR bioconductor package was used with a threshold of greater than 2-fold change and a significant false discovery rate (FDR) value of ≤ 0.001 [34]. We found a total of 12,822 transcripts with different expression between root and leaf (Additional file 1: Figure S1). Among them, 8,013 transcripts were up-regulated and 4,809 transcripts were down-regulated.

### Functional annotation of the assembled transcripts

For validation and annotation of assembled transcripts, sequence similarity searches were conducted against protein databases. BLAST searches indicated that 80,184 (45%) and 70,840 (40%) out of 177,156 transcripts showed significant similarity to known proteins in NCBI Nr and TAIR databases. As expected, the number of annotations we could assign increased when we annotated based on the closely related *Solanaceae* species of tomato and potato. Of all the transcripts, 83,204 (47%) had BLAST hits in tomato protein database, and 84,184 (47%) transcripts significantly matched proteins in the potato database.

**Figure 3:**
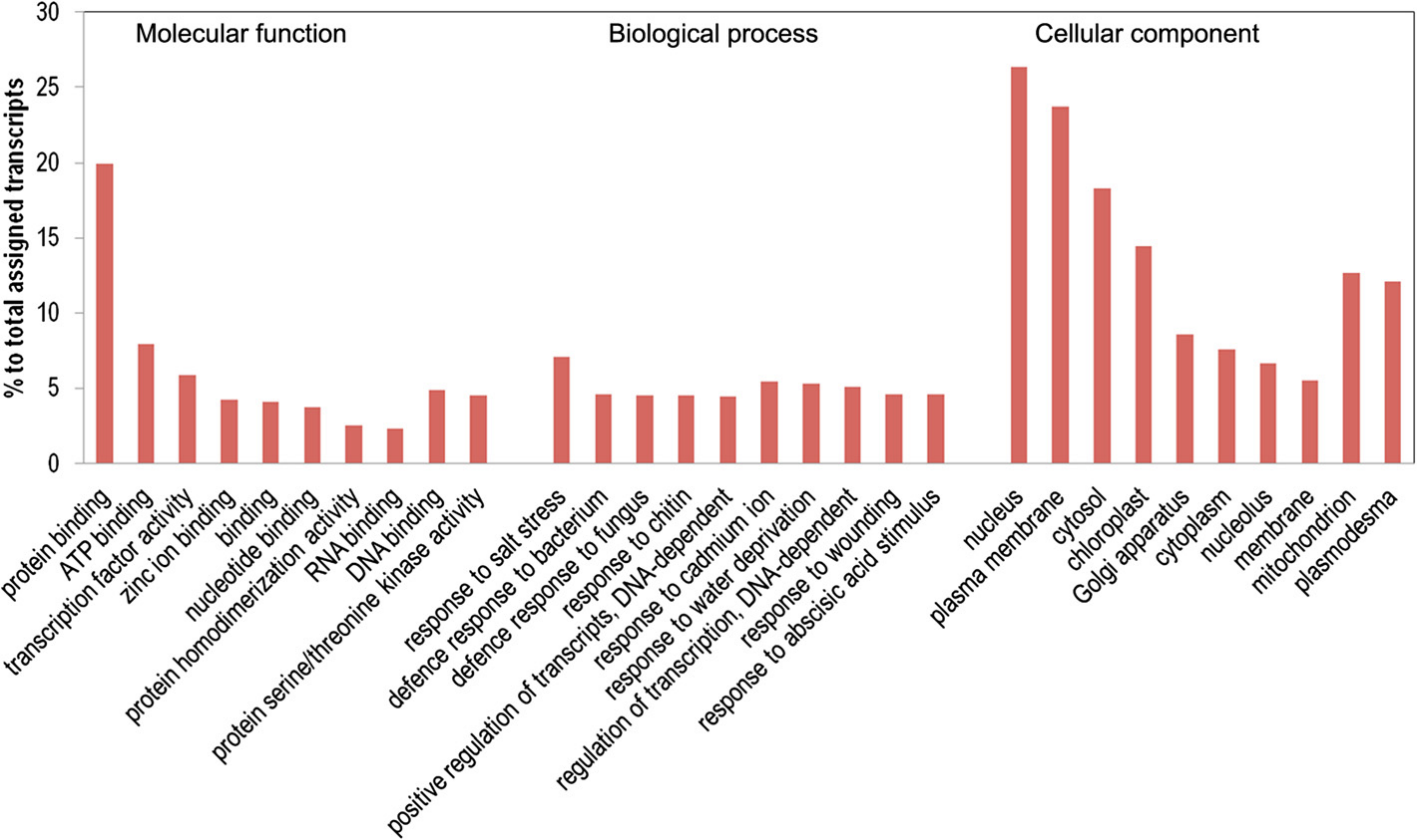
**Gene ontology (GO) classification of assembled** ***in vitro*** **tissue transcripts.** GO assignments are summarized in three main categories: Molecular function, Biological process, and Cellular component. A total of 57,404 transcripts were assigned GO terms based on BLAST matches to known proteins.

Further, gene ontology (GO) annotation [35] was carried out BLAST2GO [35] and a total of 57,404 transcripts (32.40% of all the assembled transcripts) were assigned at least one GO term. Among the GO classifications, assignments to the cellular component class ranked highest (53,140), followed by biological process (50,786) and molecular function (44,018). Within the molecular function category, transcripts assigned to protein binding (11,436) and ATP binding (4,583) processes were the most common (Figure 3). Within the biological process category, the majority of the GO terms were assigned to response to salt stress (4,067) and response to cadmium ion (3,120). For cellular components, the assignments were mostly nucleus (15,104) and plasma membrane (13,613; Figure 3).

**Figure 4:**
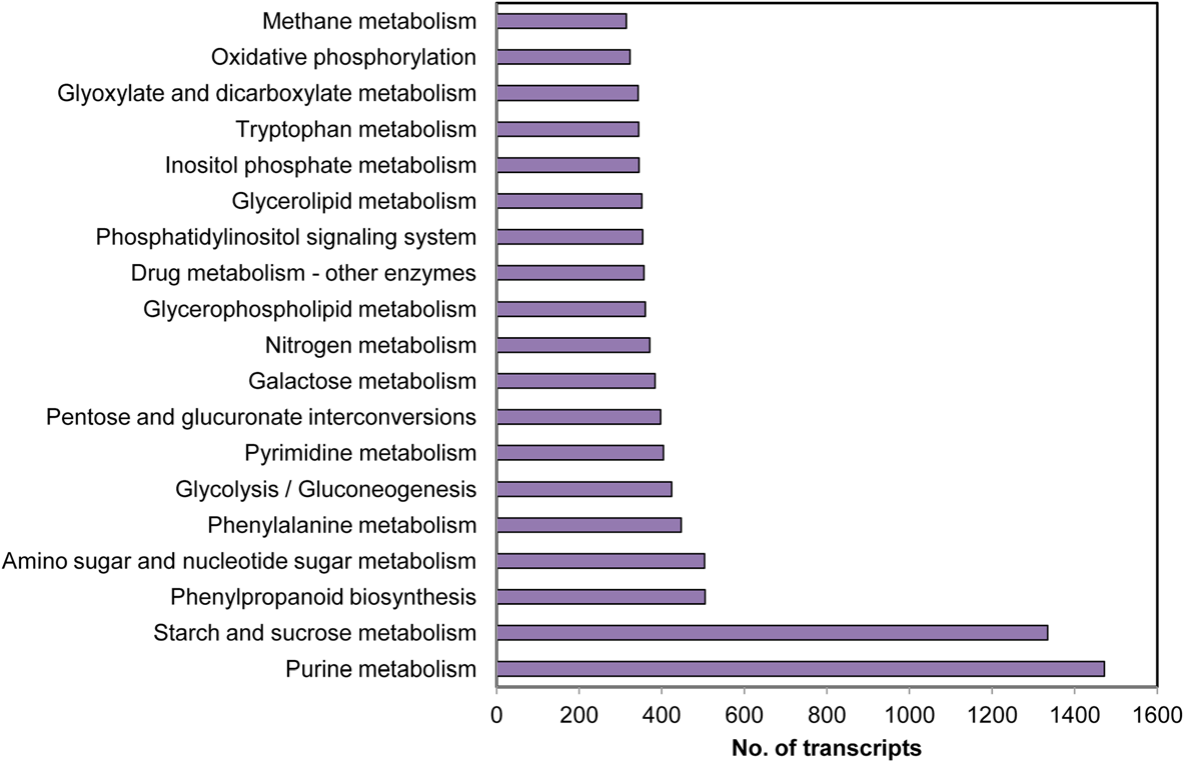
**KEGG pathway annotation of assembled** ***in vitro*** **tissue transcripts.** Pathway assignment was summarized for two main categories: metabolism and secondary metabolite categories. A total of 24,515 transcripts were assigned to primary and secondary metabolic pathways.

To identify the biological functions and interactions of genes, we used KEGG pathway [36] annotation. A total of 24,515 transcripts were assigned to 139 KEGG pathways. As shown in Figure 4, the KEGG metabolic pathways assigned most transcripts were involved in purine, starch and sucrose metabolism. Of the transcripts assigned to secondary metabolite biosynthetic pathways, a large pool with 505 members was mapped to phenylpropanoid biosynthesis (Table 2), with monoterpenoid biosynthesis (174) and terpenoid backbone biosynthesis (155) also including many transcripts. The overall annotation of the *W. somnifera* transcriptome provides a valuable resource for investigating specific processes, functional descriptions and pathways.

**Table 2:** **Differential expression of transcripts involved in major secondary metabolite biosynthetic pathways**

**Pathway**	**Pathway ID in KEGG DB**	**No. of transcripts assigned to pathway**	**Differentially expressed transcripts (more than 2 fold difference)**	**No. of unique transcripts**	
				**Adventitious roots**	**Leaves**
Phenylpropanoid biosynthesis	map00940	505	185	104	0
Phenylalanine, tyrosine and tryptophan biosynthesis	map00400	209	49	15	0
Monoterpenoid biosynthesis	map00902	174	50	23	0
Terpenoid backbone biosynthesis	map00900	155	28	22	0
Pantothenate and CoA biosynthesis	map00770	141	52	16	0
Flavone and flavonol biosynthesis	map00944	129	57	22	0
Carotenoid biosynthesis	map00906	127	47	16	0
Zeatin biosynthesis	map00908	127	43	26	0
Flavonoid biosynthesis	map00944	121	49	15	0
Ubiquinone and other terpenoid-quinone biosynthesis	map00130	103	35	13	1
Isoquinoline alkaloid biosynthesis	map00950	101	34	11	0
Tropane, piperidine and pyridine alkaloid biosynthesis	map00960	100	35	12	0
Steroid hormone biosynthesis	map00140	90	33	16	0
Various types of N-glycan biosynthesis	map00513	73	10	6	0
Steroid biosynthesis	map00100	70	11	17	0
N-Glycan biosynthesis	map00513	61	10	5	0
Folate biosynthesis	map00790	59	11	7	0
Sesquiterpenoid and triterpenoid biosynthesis	map00909	45	15	4	0
Glucosinolate biosynthesis	map00966	43	18	11	0
Diterpenoid biosynthesis	map00904	38	8	14	0
Lipopolysaccharide biosynthesis	map00540	31	8	4	0
Phosphonate and phosphinate metabolism	map00440	28	4	6	0
Other types of O-glycan biosynthesis	map00514	11	0	1	0
Isoflavonoid biosynthesis	map00943	7	5	2	0
Anthocyanin biosynthesis	map00942	6	4	1	0

We next identified differentially expressed transcripts involved in the biosynthesis of major secondary metabolites (Table 2). Differentially expressed transcripts were observed for most of the pathways analysed, which was perhaps not surprising given the large number of transcripts that were unique to *in vitro* root tissues. We mapped the 24,317 *in vitro* root-specific transcripts to KEGG pathways and observed that only 3% of the transcripts were assigned to established pathways whereas the others were of unknown function. Interestingly, 13 transcripts for beta-amyrin synthase appear to be specific to *in vitro*–grown roots as they were not found among field-grown root transcripts [20].

### Expression of major withanolide biosynthesis genes in adventitious root and leaf tissues at different culture periods

**Figure 5:**
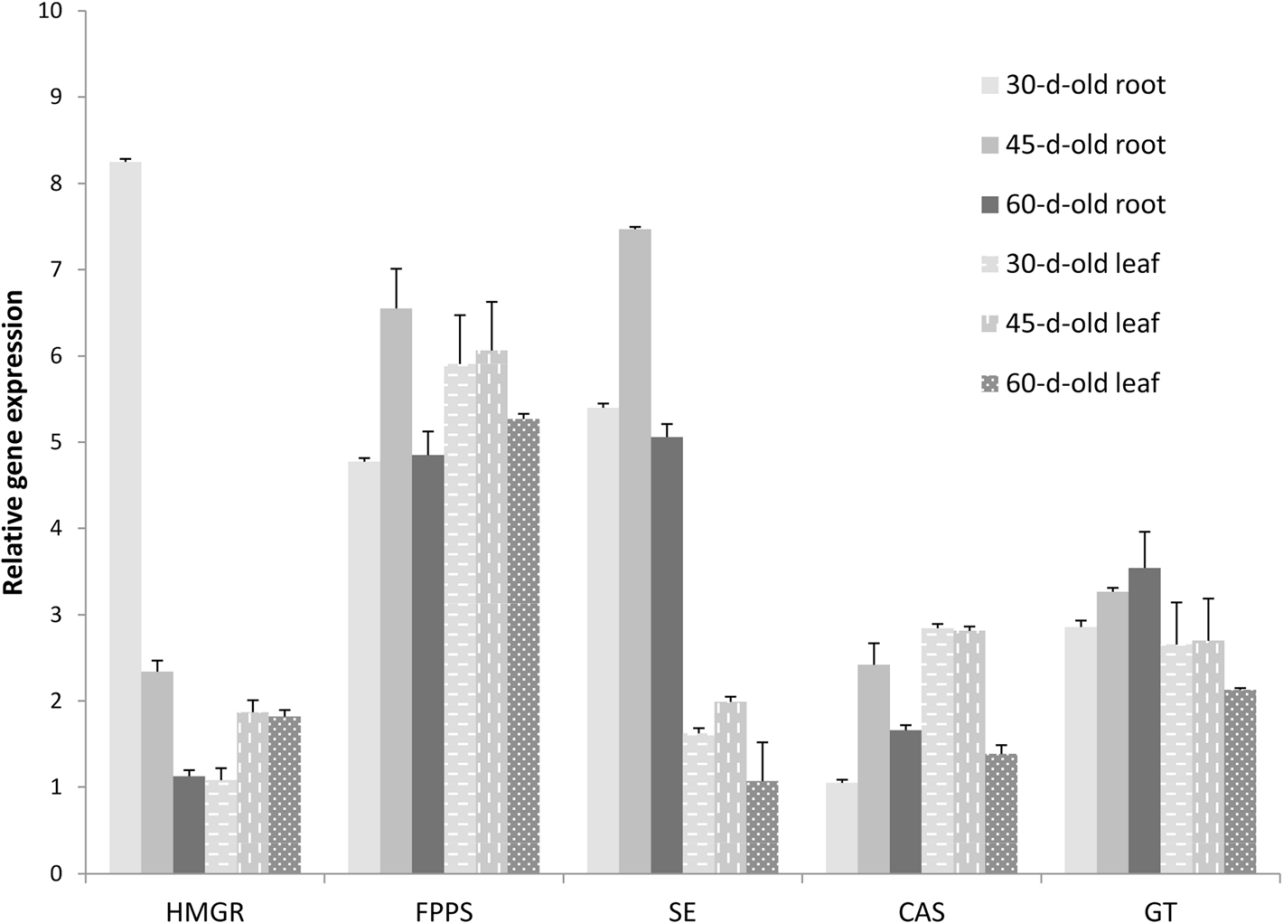
**Expression analysis of selected genes related to withanolide biosynthesis.** Adventitious roots and leaves of 30, 45, and 60 days old were collected and their total RNAs were used for qRT-PCR analysis of genes for HMGR, FPPS, SE, CAS, and GT. Relative expression levels were calculated by the 2^-ΔΔCt^ method with *GAPDH* as an internal control.

To establish a complete expression profile for genes in the withanolide biosynthesis pathway, we extracted transcripts encoding enzymes involved in withanolide biosynthesis, including FPPS, SE, CAS, DXPS, GT, and HMGR (Table 3) from our assembly. We found expression of most transcripts in both types of *in vitro* tissues, along with some *in vitro* root-unique transcripts (Table 3). There were many transcripts for methyltransferase enzymes and very few for enzymes such as CECI, MECDPS, HMBPR, HMBPS and MVD (Table 3). Overall, our data clearly showed that genes encoding putative enzymes involved in withanolide biosynthesis were expressed in both *in vitro* root and leaf tissues. For experimental confirmation, we chose major candidate genes HMGR, FPPS, SE, CAS and GT and performed qRT-PCR analysis (Figure 5). *FPPS, SE* and *CAS* genes exhibited higher expression at 45 days of growth in both leaf and root tissue. *HMGR* expression was high in 30-day-old *in vitro* adventitious root cultures, but declined sharply by the 45th day to levels observed in leaf tissues. In leaf tissue, the expression of *HMGR* was much lower, with maximal expression at 45 days of culture. *CAS* and *GT* shared similar expression levels in leaf tissues. In root tissue, *GT* levels were high until 60 days of culture, whereas in leaf tissue, *GT* exhibited similar expression during 30 and 45 days of growth but declined at 60 days. The expression of *SE* and *GT* seemed to follow a similar pattern with relatively higher expression in roots compared to leaves at all three time periods analysed, whereas *FPPS* and *CAS* shared a similar expression profile, with comparatively higher expression in leaves. The correlation matrix revealed significant linear correlation between the expression patterns of *FPPS* and *SE*, and the accumulation of major withanolides. *FPPS* and *SE* exhibited significant correlation (r = 0.56; p = 0.01) and (r = 0.75) respectively with the accumulation of withanolide A in 45-day-old root culture. In 45-day-old leaf culture, significant linear correlation (r = 0.22; p = 0.01) and a strong positive correlation (r = 0.8) were displayed by *FPPS* and *SE*, respectively, towards the accumulation of withaferin A. Among 60 day old root culture, significant correlation was exhibited by *SE* (r = 0.602 ) and *FPPS* (r = 0.699) towards the accumulation of withanolide A whereas, among 60 day old leaf culture, *FPPS* exhibited strong positive correlation (r = 0.78) followed by *SE* (r = 0.73) towards the accumulation of withaferin A. This shows that, though the roots turn brown by 60 days of culture, there is active metabolism taking place. Overall, the expression of the 5 tested genes in all time periods were comparable to the digital expression patterns observed in 60-day-old *in vitro* root and leaf tissue sequencing data based on FPKM values (Additional file 2: Figure S2).

**Table 3:** **Number of transcripts found in** ***in vitro*** **roots and leaves putatively encoding enzymes involved in withanolide biosynthesis**

**Enzymes**	**Abbreviation**	**Enzyme commission number**	**No. of transcripts**		
			**Total**	**Expressed in both adventitious roots and leaves**	**Uniquely expressed in adventitious roots**
1-Deoxy-D-xylulose-5-phosphate synthase	DXPS	2.2.1.7	8	8	0
1-Deoxy-D-xylulose-5-phosphate reductoisomerase	DXPR	1.1.1.267	3	3	0
2-C-Methyl-D-erythritol 4-phosphate cytidylyltransferase	MEP-CT	2.7.7.60	2	2	0
4-(Cytidine 5′-diphospho)-2-C-methyl-D-erythritol kinase	CDP-MEK	2.7.1.148	3	2	1
2-C-Methyl-D-erythritol 2,4-cyclodiphosphate synthase	MECDPS	4.6.1.12	1	1	0
(E)-4-Hydroxy-3-methylbut-2-enyl-diphosphate synthase	HMBPPS	1.17.7.1	1	1	0
4-Hydroxy-3-methylbut-2-enyl diphosphate reductase	HMBPPR	1.17.1.2	1	1	0
Isopentenyl-diphosphate delta-isomerase	IDI	5.3.3.2	4	4	0
Acetyl-CoA C-acetyltransferase	ACAT	2.3.1.9	19	12	7
Hydroxymethylglutaryl-CoA synthase	HMGCS	2.3.3.10	5	5	0
Hydroxymethylglutaryl-CoA reductase	HMGR	1.1.1.88	11	11	0
Hydroxymethylglutaryl-CoA reductase	HMGR	1.1.1.34	9	9	0
Mevalonate kinase	MK	2.7.1.36	3	3	0
Phosphomevalonatekinase	PVMK	2.7.4.2	8	8	0
Diphosphomevalonate decarboxylase	MVD	4.1.1.33	1	1	0
Geranyl-diphosphate synthase	GPPS	2.5.1.1	38	35	3
Farnesyl-diphosphate synthase	FPPS	2.5.1.10	19	19	0
Squalene synthase	SS	2.5.1.21	5	5	0
Squalene monooxygenase	SE	1.14.13.132	10	10	0
Cycloartenol synthase	CAS	5.4.99.8	28	15	13
Sterol 24-C-methyltransferase	SMT1	2.1.1.41	7	7	0
Methylsterol monooxygenase/Sterol-4a-methyl oxidase 2	SMO1/SMO2	1.14.13.72	7	6	1
Cycloeucalenol cycloisomerase	CEC1	5.5.1.9	1	1	0
Obtusifoliol 14-demethylase	CPY51G1	1.14.13.70	3	3	0
Delta 14-sterol reductase	FK	1.3.1.70	3	3	0
C-7,8 Sterol isomerase	HYD1	5.3.3.5	2	1	1
C-5 Sterol desaturase	STE1	1.14.21.6	12	10	2
7-Dehydro cholesterol reductase	DWF5	1.3.1.21	2	2	0
Sterol glycosyltransferases	SGT	2.4.1.173	53	42	11
Methyltransferases	MT		509	466	43

### Quantification of major withanolides in *in vitro* leaf and root tissues

**Figure 6:**
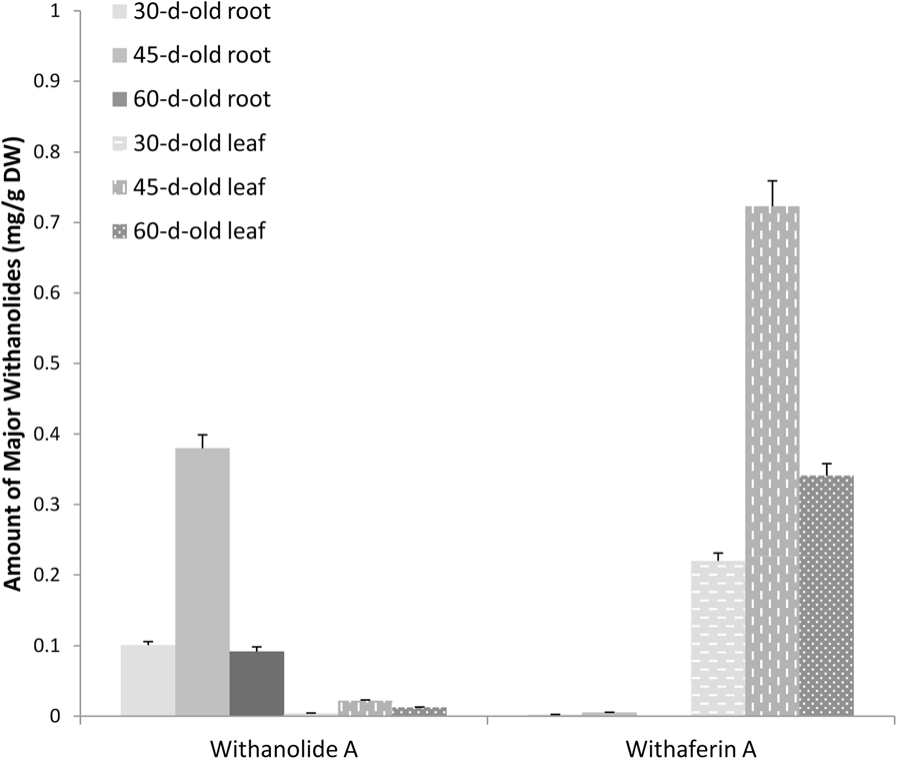
**Accumulation of major withanolides in** ***in vitro*** **grown samples.** Withanolide contents in *in vitro* adventitious root and shoot cultures (after 30, 45 and 60 days of growth) quantified using HPTLC. The data are the mean ± SE of three replicates obtained from two independent experiments.

The major secondary metabolites of *W. somnifera,* namely withanolide A and withaferin A, were quantified by high performance thin layer chromatography [37] (HPTLC; Figure 6). The contents of both components were significantly different in the two tissues tested (Additional file 3: Figure S3). Leaf tissues preferentially accumulated withaferin A whereas *in vitro* adventitious root tissues accumulated prodigious quantities of withanolide A. Significant differences were also observed in the accumulation patterns over time. *In vitro* adventitious roots contained high amounts of withanolide A (380 ± 0.36 μg/g DW) at 45 days, but less at 60 days of culture. Similarly, withaferin A accumulation (980 ± 0.97 μg/g DW) in leaf tissue was highest at 45 days and reduced on extended culturing period.

### Antioxidant activity of extracts of *in vitro* leaf and root tissues

Complex antioxidant systems are essential for protecting cellular membranes and organelles from the damaging effects of reactive oxygen species (ROS), and plants have developed a series of enzymatic and non-enzymatic detoxification systems to counteract ROS [38]. The enzymatic and non-enzymatic antioxidant activities of the cultured *W. somnifera* tissues is represented in Table 4. Though both tissues were grown under controlled conditions, the antioxidant potentials differed between extracts of *in vitro* adventitious root (root extract) and leaf (leaf extract). The antioxidant capacity, as estimated from total antioxidants, of root extracts was much higher than that of leaf extracts (Table 4).

**Table 4:** **Antioxidant activity in methanolic extracts of adventitious root and leaves**

**Activity of antioxidants**	**Tissue**	
	***In vitro*** **adventitious root**	***In vitro*** **leaf**
**Enzymatic antioxidants (U/mg protein)**		
SOD	5.66 ± 0.35	3.38 ± 0.25
Peroxidase	3.02 ± 0.42	2.5 ± 0.57
GST	4.0 ± 0.25	2.4 ± 0.09
Polyphenol oxidase	3.50 ± 0.23	2.56 ± 0.35
**Non-Enzymatic antioxidants (mg/g)**		
Vitamin A	3.54 ± 0.35	2.56 ± 0.25
Vitamin C	3.8 ± 0.27	1.47 ± 0.46
Vitamin E	10.2 ± 0.27	8.3 ± 0.57
Free radical scavenging activity (FRSA)	72.5 ± 0.58%	55 ± 0.47%

The values of Mean ± SD of triplicates.

SOD (U/mg): amount of SOD that causes 50% reduction in the extent of NBT oxidation.

Peroxidase (U/mg): change in absorbance/min at 430 nm.

GST (U/mg protein): moles of CDNB conjucated/min/mg of sample.

Polyphenol oxidase: (U/mg protein): change in absorbance/minute at 495 nm.

### Inhibitory effect of methanolic extract of *in vitro W. somnifera* leaf tissue on cell proliferation

**Figure 7:**
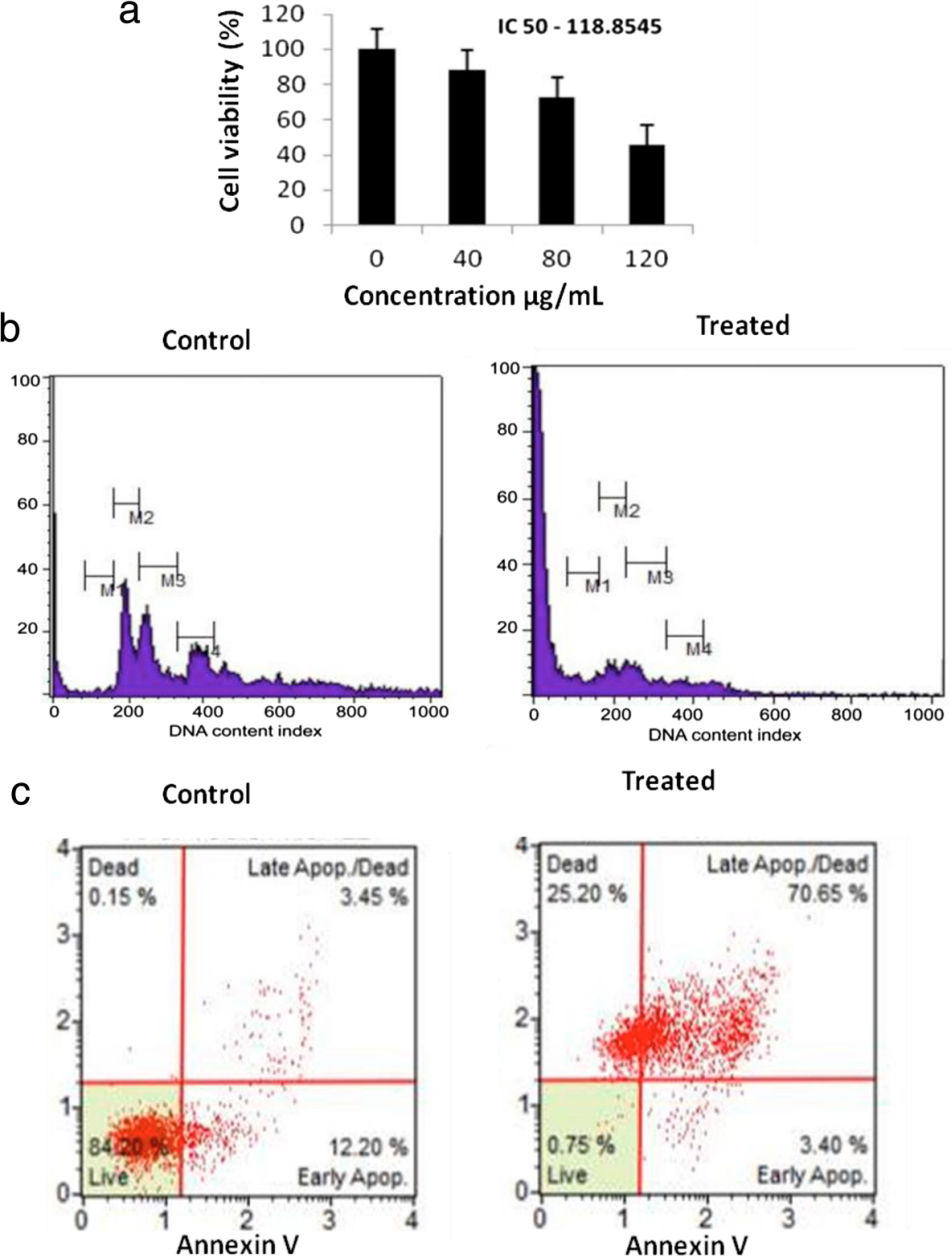
**Influence of i** ***n vitro*** **leaf extract on AGS cell growth inhibition. a**- Inhibitory effect of *in vitro* methanolic leaf extract on AGS cell proliferation was measured by MTT assay. IC-50 value of the extract on the cell viability was identified as 118.85 μg/mL. **b** – DNA cell cycle analysis of control and treated AGS cells. Cells were stained with PI to determine cell cycle phase distribution based on fluorescence of DNA. **c** - Apoptosis level was examined by flow cytometry for control and treated AGS cells. Cells were treated with methanolic extract of *in vitro* grown leaf tissue (IC50 - 118 μg/mL) for 24 h. Data are representative of three similar experiments.

An initial cytotoxicity study based on the survival of adenocarcinoma gastric cancer (AGS) cell lines was performed using an MTT (3-(4, 5-dimethylthiazolyl-2)-2, 5-diphenyltetrazolium bromide) assay. Cells were exposed to various concentrations (0–200 μg/mL) of methanolic extract of *in vitro*-grown leaf tissues (leaf extract) and the cell viability was determined. Cytotoxicity was quantified as the percentage of viable treated cells relative to the viable cells in an untreated control. The half maximal inhibitory concentration (IC_50_) for leaf extract was 118.85 μg/mL (Figure 7a). To determine the amount of sub G_0_/G1 cells present, the AGS cell lines were treated with leaf extract at the maximal IC_50_ concentration (118.85 μg/mL). The cells were stained with propidium iodide (PI) and analyzed using flow cytometry. Statistically significant accumulation of cells with sub-G1 DNA content and other remarkably detectable cell cycle changes were noted after 24 h incubation. Consistent with an increased sub-G1 AGS cell population, there was a notable decrease of cells in G_1_, S and G_2_/M phases. The control cells that were treated with phosphate buffer saline (PBS) alone showed no changes in cell cycle regulation (Figure 7b). Annexin V/PI staining followed by flow cytometry was used to confirm the effect of apoptosis on AGS cells. Treatment of cells with methanolic extract of *in vitro*-grown leaf tissue resulted in more apoptotic cells, and the percentage of cells in late apoptosis was much higher than that in early apoptosis (Figure 7c). These data suggest that leaf extract from *in vitro-*grown *W. somnifera* inhibits AGS cell proliferation by inducing cell cycle arrest and apoptosis.

### Comparative analysis of *in vitro-* and field-grown leaf and root transcripts

To investigate tissue or culture method-unique gene expression, we compared our 60-day-old *in vitro* leaf and root transcripts with one-year old field-grown root and leaf sequences that were downloaded from the NCBI SRA database (accession no. SRR520143) [20]. We selected high-quality reads from *in vitro* root and leaf samples and assembled them separately using Trinity. This assembly yielded a total of 160,323 transcripts with an average length of 1,028 bp for root and 173,009 transcripts with an average length of 1,060 for leaf. Then, we mapped field-grown root sequences onto our *in vitro* root transcripts with a minimum alignment length of 200 bp. Only 5.7% of the transcripts were not found in field-grown roots, and we consider these transcripts as *in vitro* root-specific transcripts. Similarly, field-grown leaf sequences were aligned onto our *in vitro* leaf data set, revealing about 3.9% of *in vitro* leaf-specific transcripts.

Furthermore, we assembled field-grown leaves and root tissues separately using CLC assembler to identify field-grown specific transcripts. We did not find a significant number of field grown tissue-specific transcripts. This implies that our assembly from cultured tissue contains a large enough number of contigs to avoid much loss of information.

### Identification of cDNA-derived EST-SSR markers

Transcript-based SSR markers are important resources for genetic diversity analysis, marker-assisted selection, and genetic mapping. For identification of SSRs, *W. somnifera* Nr set transcripts were searched with perl script MISA (http://pgrc.ipk-gatersleben.de/misa/). The following criteria for SSR identification were used in the MISA script: mono-nucleotides repeated more than ten times, di-nucleotides repeated more than six times, and tri-, tetra-, penta- or hexa-nucleotides repeated more than five times. A total of 16,283 potential EST-SSRs were identified in 13,944 transcripts, of which 1,951 sequences contained more than 1 EST-SSR and 678 EST-SSRs were present in compound form. The di-nucleotide SSRs represented the most abundant type (2,489) of SSRs identified followed by tri-nucleotide (1,681) SSRs. We also identified tetra (92)- and penta (20)-nucleotide SSR motifs. Although only a small fraction of tetra- and penta-SSRs were identified in *W. somnifera*, the number is quite significant and will provide a cost-effective method for development of functional genetic markers in *W. somnifera*.

### Withanomics database

We developed a public data resource, Withanomics, which provides a searchable interface for the *W. somnifera* transcriptome data. Withanomics is freely available at http://im-crop.snu.ac.kr/withania/index.php. The current version of this database provides the total transcriptome sequences (root + leaf) and leaf (*in vitro*) sequences and root (*in vitro*) sequences separately reported in this study, as well as EST sequences produced from our previous study [30]. Expression data for transcripts can be accessed using unique transcript IDs which report the expression of the transcripts in root and leaf tissues. Functional searches for all transcripts can be carried out using functional keywords with the annotation database. Additionally, we included an *in vitro* root-specific function option to extract function of *in vitro* root-specific transcripts. The database uses the NCBI BLAST algorithm (version: 2.2.15) for sequence-based searches. From BLAST searches, users can match nucleotide or protein sequence(s) against the *W. somnifera* whole transcriptome data, separate tissue transcriptome data, and ESTs from root and shoot data at user-defined parameters. Furthermore, we developed a sequence extract tool to extract or download single or bulk sequences from our database. We anticipate that the database will be useful for accelerating genomic research in *Withania* and we will be updating this database annually or as new sequence data sets are available for *Withania*.

## Discussion

### Establishment of *in vitro* cultures and transcriptome sequences of *W. somnifera*

*W. somnifera* is a medicinal plant of high therapeutic value, and withanolides are the major secondary metabolites responsible for most of the pharmacological activity of this plant. The accumulation of secondary metabolites in field-grown plants can be influenced by various environmental factors as well as the age of the plant at harvest [39]. The growing demand in the pharmaceutical industry and the lack of homogeneity in field-grown material led us to search for an alternative approach for mass production of *W. somnifera* roots. Adventitious *W. somnifera* roots grown *in vitro* have been reported to produce the pharmaceutically important withanolides, especially withanolide A [8,40-42]. Root tips are auxin-accumulating regions where active cell multiplication takes place, and hence were chosen for establishment of suspension cultures. Leaves from shoots devoid of roots were chosen from *in vitro* multiplied plants. This was done to ensure that the transcriptome library we constructed would represent transcripts exclusively specific to root or leaf tissues. *In vitro* cultures have been successfully exploited for the production of paclitaxel, an anticancer agent from *Taxus* species, at industrial scale, and attempts are being made to increase production using elicitors [43]. However, only when bottlenecks in the biosynthetic pathways have been identified will it be possible to overexpress the genes that control the rate-limiting steps and thus boost the production of valuable secondary metabolites in *in vitro* cultures.

In our current study, we generated a total of 249 million high-quality reads from *in vitro*-grown leaf and root tissues, more than the previously reported ESTs and transcriptome data [20,30,31]. We obtained a total transcriptome size of 183 Mbps with average transcript length of 1,033 bp (Table 1) which is comparatively better than a previous *Withania* 454 transcriptome study [20]. Significant differences in the number of transcripts annotated previously [20] and in the present study were documented. Functional genomics studies require highly reliable reference sequences and our assembly data can be utilized as such for *W. somnifera* due to the high sequencing depth. GO annotation showed that many transcripts were assigned to regulation-related process such as response to stress and wounding and defence responses in the biological process category. Transcripts related to GO term nucleus were most abundant in the cellular component category, which is similar to the previously reported GO annotation of field-grown root and leaf tissues [20]. Further, pathway assignment for all transcripts was performed based on the KEGG database. Previously, Gupta *et al.* [20] identified a total of 124 pathways among one-year-old field-grown leaf and root tissue transcripts, while 139 pathways were identified in the present study. In both studies, the phenylpropanoid biosynthesis pathway was found to harbour the largest cluster of transcripts. In a recent study, Dasgupta *et al.* [31] reported 184 pathways from their *in vitro* leaf transcriptome library. The additional pathways may be due to pathogenesis-related genes expressed in response to salicylic acid signalling in the source tissue of their study.

### Identification and expression of genes related to withanolide biosynthesis

With respect to the genes involved in withanolide biosynthesis, we observed all genes to be expressed in both roots and leaves, similar to the results of Gupta *et al.* [20]. As a part of our ongoing endeavour, we validated important withanolide pathway genes including *HMGR, FPPS, SE, CAS* and *GT* at different time periods of culture (Figure 5). HMGR catalyses the reduction of HMG-CoA to mevalonate, reported to be the first committed step in the isoprenoid pathway [44]. FPPS catalyses the biosynthesis of FPP, which serves as a substrate for the first committed reaction of several branched pathways [45]. *SE* and *CAS* genes comprise vital genetic components of biosynthetic pathways leading to production of phytosterols and a large cluster of triterpenoids [46]. GT performs the glycosylation of a wide variety of natural compounds, which is an important mechanism in detoxification of exogenous compounds [47].

Among these genes, the expression of *HMGR* was notable in that it showed very high expression during the 30th day of culture in *in vitro* roots. Differences in expression of *HMGR* could be essential to accommodate the changing demand for a cellular array of isoprenoids during normal growth and development of the plant, as is evidenced by the tissue-specific expression of members of this multi-gene family in several plant species [48]. *HMGR* has been reported to be present in two copies in *Arabidopsis thaliana* [49] and three to four copies in *Solanum* species [48]. Expression of *HMGR* varies in a time-dependent manner in *in vitro* cultures of *Dioscorea zingiberensis* [50], which is similar to our observation. FPPS is another of key enzyme in the isoprenoid biosynthetic pathway, wherein it initiates triterpenoid precursor biosynthesis to support withanolide production [51]. Any change in the levels of FPPS would alter the flux of isoprenoids towards various branches of this pathway, playing a crucial role in isoprenoid metabolism. CAS plays essential roles in plant cell viability and in the regulation of triterpenoid biosynthesis. Upregulation of CAS results in enhanced accumulation of diosgenin in *in vitro* cultures of *D. zingiberensis* [50]*.* In general, the expression of *FPPS* and *CAS* were found to be higher in leaves at all time points in *in vitro* shoots compared to roots, which is in accordance with previous observations [51,8].

SE is a noncytochrome-P450 type monooxygenase that catalyses the formation of a hydroxyl group that is characteristic of sterols and triterpenols and represents a rate-limiting step in sterol biosynthesis [52-54]. A higher level of *SE* expression was observed in *in vitro* roots than in leaf tissues, consistent with a previous report of higher expression of *SE* in one-year-old field-grown roots than in shoot tissues of *W. somnifera* [8]. Glucosylation of sterols is catalyzed by sterol glucosyltransferases (SGTs), which are family 1 glycosyltransferases. The expression of *SE* and *GT* was found to be higher in *in vitro* roots at all time periods compared to leaves. Among the time periods analysed, all genes investigated except *HMGR* showed the highest expression at the 45th day of culture.

In the present study, the high expression of *SE* observed in root tissue was in corroboration with the data of Sabir *et al.* [8], who reported a correlation between high expression of the genes for squalene epoxidase and squalene synthase and relatively higher accumulation of withanolide in field-grown root tissues. *SE* was reported to be the rate-limiting enzyme in sterol biosynthesis [52-54], and this could explain the high withanolide accumulation in 45-day-old *in vitro* root culture. Though expression of *FPPS* was higher in *in vitro* root tissue at 45 days, *WsFPPS* may not modulate withanolide content [51] as FPP is also reported to be involved in biosynthesis of triterpenoids, carotenoids, plastoquinones and many other molecules. Next to *FPPS*, expression of *CAS* was highest in root tissues. After the formation of epoxy squalene, CAS catalyzes the next step, which involves the formation of 24-methylene cholesterol, a precursor molecule for withanolide synthesis. Hence, expression of *CAS* also plays a significant role in the biosynthesis of withanolides.

We can surmise that the key pathway genes are expressed to different levels in different parts of the plant thus showing tissue-specific expression. This variation may be because of the presence of specific trans-acting factors required for their functional activation. Expression analysis of other genes involved in the biosynthetic pathway along with inhibitor studies will aid in enhancing the specific accumulation of desirable secondary metabolites.

### Pattern of withanolide accumulation

It has been hypothesised that withanolides are produced in different parts of the plant through expression of the metabolic pathway rather than being imported from leaves [41]. Our current results support this hypothesis in that the expression of important genes involved in withanogenesis showed a differential pattern along with the accumulation of important withanolides, namely withanolide A and withaferin A. The accumulation of both withanolide A and withaferin A was found to be high during 45 days of culture, but interestingly, accumulation of withanolide A in leaves was nearly absent as was that of withaferin A in roots.

This observation is contradictory to the previous reports of Sabir *et al.* [8], but is in accordance with the observation of Dasgupta *et al.* [31] who reported a low concentration of withanolide A in control leaf samples, which are comparable to the 45-day-old leaves in the present study. The accumulation of withanolide A in roots followed a similar pattern to the previous report [8], where withaferin A was absent in *in vitro* root cultures. Thus, withaferin A was found to be tightly linked to leaf tissue indicating that there was no coordination of the accumulation profile between root and leaf tissue. The results of the present study are particularly important as they indicate that the *in vitro*-cultured roots and shoots possess *de novo* withanogenic competence and that *in vitro* cultures can be manipulated for efficient production of specific withanolides.

### Antioxidant efficacy of *in vitro* tissues

We found that the *in vitro*-cultured roots of *W. somnifera* are good source of both non-enzymatic and enzymatic antioxidant components. *In vitro*-cultured root tissue showed high SOD and GST activity in addition to polyphenol oxidase and peroxidase activity. Present in all subcellular organelles, SOD serves as the first line of defence against free radicals. GSTs are most often thought of as detoxification enzymes and can metabolize a wide range of toxic exogenous compounds via GSH conjugation [55]. Polyphenol oxidase is localized in the chloroplast and is a copper-associated enzyme with two binding sites for phenolic substrates. Previous research reported that polyphenol oxidase is activated only when the plant undergoes physical damage [56]. This might explain the relatively low level of polyphenol oxidase activity in *in vitro* root and leaf tissue. Increased peroxidase activity is associated with environmental stress on plants [57]. The low peroxidase activity in *in vitro* cultures could result from a related lack of expression of the enzyme. Among the non-enzymatic antioxidants, vitamin E was found to be the most abundant in *in vitro* root cultures. Vitamin E is a lipid-soluble vitamin found in cell membranes and circulating lipoproteins that offers protection against oxidative damage by acting directly with a variety of oxygen radicals. Its antioxidant function is strongly supported by regeneration promoted by vitamin C [58]. Hence, these results support the view that *in vitro*-cultured roots are promising source of potential antioxidants and function similar to field-grown tissues.

Combining the promising evidence that the *in vitro* leaf tissues express higher quantities of withaferin A than the *in vitro* root tissues, we proceeded to analyse the cytotoxicity of *W. somnifera* leaf extract on AGS cell lines. We found that the samples had the ability to linearly produce 50% cell death with the maximally effective concentration being 100–120 μg/mL. The effect of *W. somnifera* leaf extract on AGS cancer cells establishes that tissues grown *in vitro* are capable of inducing apoptosis and regulating the cell cycle of DNA biosynthesis. It has been previously reported that withaferin A, a promising anticancer constituent of *W. somnifera*, inhibits growth of human breast cancer cells in culture and *in vivo* in association with apoptosis induction [59]. Overall, the apoptosis and cell cycle analysis in cancer cells demonstrates that the *in vitro W. somnifera* leaves have anticancer effects, especially on human gastric adenocarcinoma cell lines.

## Conclusion

Here, transcriptome profiles for *in vitro*-cultured leaf and root tissues of *W. somnifera* provided substantial information with respect to expression of genes involved in withanolide biosynthesis and accumulation of major withanolides. We have also shown that accumulation of different metabolites is linked to tissue type and that the extracts of *in vitro* root and leaf tissues have antioxidant potential and anti-proliferative capacity against an AGS cancer cell line, respectively. Further, we developed a user-friendly public database to accelerate research. Altogether, our research provides valuable information on the *W. somnifera in vitro* transcriptome and withanolide biosynthetic pathway gene expression to promote effective engineering of the pathway genes for improved biosynthesis of withanolides.

## Methods

### Plant material and RNA extraction

Seeds of *W. somnifera* (L.) Dunal ‘Jawahar Asgandh 20’ were obtained from the Central Institute of Medicinal and Aromatic Plants (Lucknow), and germinated *in vitro* on MS solid basal medium supplemented with 2% sucrose in the dark at 25°C. Shoots from *in vitro*-germinated seedlings were maintained on MS basal medium under standard culture conditions. Shoots were multiplied on MS medium supplemented with 1 mg/L BAP, with regular subcultures. Multiplied shoots were transferred to hormone-free medium and maintained. Leaves from these shoots devoid of roots were used for RNA extraction. Adventitious roots were induced from leaf explants following the procedure described by Wasnik *et al.* [60]. Direct adventitious roots were induced from leaf explants of *W. somnifera* cultivar ‘Jawahar Asghand-20’ on Murashige and Skoog (MS) medium supplemented with 30 g/L sucrose, 1 mg/L IBA and 0.25 mg/L IAA. For mass culture of adventitious roots, the root tips and branches from *in vitro*-induced adventitious roots were transferred to liquid MS basal medium (Figure 1) in a bubble column bioreactor (Biopia, Korea). RNA was extracted from adventitious root and leaf tissues grown on MS medium following standard protocols. The tissues were submerged in liquid nitrogen, snap frozen and crushed using a mortar and pestle. Total RNA was extracted from the frozen tissues using TRIzol reagent (Bangalore genei, India) according to the manufacturer’s instructions. Briefly, 1.5 mL TRIzol was added to 200 mg tissue, homogenized and centrifuged. The supernatant was mixed with 0.2 mL chloroform and incubated at −20°C for 20 min. The tubes were then vortexed and centrifuged, and the aqueous phase was mixed well with 0.2 mL 2-propanol followed by centrifugation. All centrifugations were carried out at 4°C, 7200 × g for 20 min. Following centrifugation, the supernatant was discarded, and the RNA pellet was washed with 75% ethanol and vortexed. Samples were again centrifuged at 7200 × g for 10 min at 4°C. After removal of the supernatant, the remaining ethanol was allowed to evaporate. The total RNA was re-suspended in diethylpyrocarbonate (DEPC)-treated water.

### Illumina sequencing and quality control

Raw reads (101 bp PE) were generated for 60-day-old *in vitro*-cultured adventitious root and leaf tissues without replication using the Illumina Hiseq2000. The library construction and sequencing was performed by the Macrogen (http://dna.macrogen.com/, Korea). The sequence data generated in this study have been deposited at NCBI in the Short Read Archive (SRA) with accession number SRP040231. The sequencing reads were cleaned by removing low-quality reads, primer adaptor sequence using NGS QC Toolkit [61]. De novo assembly was performed using Trinity [32] and to eliminate mis-assemblies and ensure a collection of informative transcripts, read depth analysis was carried out. The read depth percentage was determined by dividing the number of mapped reads for each assembled transcript by the effective length (without ‘N’) of the transcript. Transcripts with <1% read-depth were removed as those were not considered to be assembled correctly from paired reads.

### Functional annotation

Sequence similarity searches were conducted against the NCBI non-redundant (Nr) database, tomato (http://solgenomics.net/organism/Solanum_lycopersicum/genome; version ITAG2.3), potato (http://solgenomics.net/organism/Solanum_tuberosum/genome), and TAIR (The Arabidopsis Information Resource) protein databases using the BLASTx algorithm with an *E* value threshold of 10^−5^. The BLAST2GO program was used to obtain GO terms for all assembled transcripts [35]. In many cases, multiple terms were assigned to the same transcripts, and the GO terms were classified into biological process, cellular component, and molecular function.

### Quantitative reverse-transcription PCR (qRT-PCR) analysis

For expression analysis of pathway genes, adventitious root and leaf tissues were collected after 30, 45 and 60 days of culture for total RNA isolation using TRIzol reagent (Bangalore genei, India) [62]. Extracted RNA was treated enzymatically with DNase I to remove contaminant genomic DNA. Quality of RNA was examined in 1.2% denaturing formaldehyde agarose gels, and the concentration was determined using a spectrophotometer. cDNA synthesis was carried out from 2 μg RNA template using Superscript II reverse transcriptase (Invitrogen, USA), according to the manufacturer’s instructions. qRT-PCR was performed in a realplex^2^ Mastercycler (Eppendorf, Germany) in a 10-μL reaction volume containing 100 ng cDNA template with Luminoct SYBR® Green Master Mix (Sigma Aldrich, USA) and gene-specific primers. Specific primers were designed using the software primer3 [63] (Additional file 4: Table S1). The primers for *GAPDH* were used as a control to ensure that equal amounts of cDNA were used in all reactions. The PCR conditions were as follows: predenaturation at 95°C for 5 min, 40 cycles of 95°C for 30 s, 60°C for 30 s, 72°C for 30 s. The increase in fluorescence corresponding to the exponential increase in the product was used to determine the threshold cycle (C_t_) in each reaction. Relative expression levels were calculated by the 2^-ΔΔCt^ method [64] with *GAPDH* as an internal control. All qRT-PCR reactions were performed in triplicate.

### Quantification of major withanolides

Total withanolides from 1.0 g *in vitro* adventitious root and leaf tissues were extracted as described previously [65]. The final extraction was done in methanol, and the samples were evaporated to dryness, dissolved in 5.0 mL HPLC-grade methanol and subjected to HPTLC analysis. The methanolic extracts of the *in vitro* adventitious root and *in vitro* leaf (root extract and leaf extract, respectively) tissues were applied to the plates as 6-mm bands, under a stream of nitrogen, by means of a Linomat V semiautomatic sample applicator (CAMAG, Switzerland) fitted with a 100-μL syringe. Linear ascending development to a distance of 8 cm was carried out on a twin-trough chamber saturated with the mobile phase, toluene: ethyl acetate: formic acid (5: 5: 1). Subsequent to development, the banding patterns were visualized in 254 nm, 366 nm and white light and the R_f_ values were calculated. Densitometric scanning was performed with a Scanner III (CAMAG, Switzerland) in the reflectance-absorbance mode at 234 nm for withanolide A and 223 nm for withaferin A [37]. Concentrations of the compound chromatographed were determined from the intensity of diffusely reflected light. Evaluation was carried out by comparing peak areas with linear regression [66]. Pearson’s correlation coefficient (r) was used to correlate the expression levels with metabolite accumulation in 45-and 60 day-old culture in bivariate linear correlations.

### Analysis of antioxidant activity

Total enzymatic and non-enzymatic antioxidant activity in methanolic extracts of 60-day-old adventitious root and leaf tissues were analysed following standard protocols. Total antioxidant activity was assayed using DPPH scavenging activity as described by Ansari *et al.* [67] and ascorbic acid was used as reference material. For the enzymatic antioxidants, the superoxide dismutase assay was performed following Beauchamp and Fedovich [68], that for polyphenol oxidase according to Ensiminger [69], peroxidase according to Malik and Singh [70] and glutathione S transferase according to Habig *et al.* [71], and controls were run simultaneously without the plant extract. Among the non-enzymatic antioxidants, contents of ascorbic acid, vitamin A, and α-tocopherol were determined following the procedure of Roe and Kuether [72], Bayfield and Cole [73], and Backer *et al.* [74], respectively. The complete assay mixture without the plant extract served as the control to monitor non-specific binding of the substrates.

### Cell proliferation analysis using MTT assay

The capacity of methanolic extracts of 60-day-old leaves, harvested from shoots maintained *in vitro,* to prevent proliferation of AGS cells was determined by performing 3-(4, 5-dimethylthiazol-2-yl)-2, 5-diphenyltetrazolium bromide (MTT) assay. AGS cells were seeded at 10 × 10^4^ cells/mL in a 12-well plate and incubated for 24 h. The plant extracts were evaporated to dryness and dissolved in PBS before performing the experiment. The cells were treated with various concentrations of methanolic extracts of *in vitro* grown leaf tissues and incubated for 24 h. The cells treated with PBS alone (0 μg/mL) were considered to be the control with 100% viability. After incubation, 100 μL MTT solution (5 mg/mL in 1× Phosphate Buffered Saline, PBS) was added to the wells and incubated for 3 h. Then 500 μL DMSO was added to each well after complete removal of the medium to dissolve the cellular crystalline deposits and kept for 20 minutes. The optical density was measured at 540 nm using an ELISA plate reader (EIA plate, USA).

### Cell cycle analysis using propidium iodide

AGS cells were seeded in 5-mL plates at a concentration of 60×10^4^ cells/well and incubated overnight at 37°C in an atmosphere of 5% CO_2_. The cells were treated with various concentrations of methanolic extracts of *in vitro-*grown leaf tissues and incubated for 24 h. Cells were trypsinized, washed twice with cold PBS, and centrifuged. The pellet was fixed using cold 70% ethanol for 30 minutes at 4°C. The cells were washed once with PBS and resuspended in cold PI solution (50 μg/mL) containing RNase A (0.1 mg/mL) in PBS for 30 min in dark. In a Fluorescence Activated Cell Sorting (FACS) Calibur apparatus (Becton Dickinson, San Jose, CA, USA), approximately 10,000 counts were done for each sample. The relative proportions of G0/G1, S and G2/M cells were determined for cell cycle analysis. By determining the percentage of cell distribution in each phase, the effect of leaf extract on cell cycle was measured.

### Cell apoptosis analysis

Annexin V/PI staining was employed to classify apoptosis levels in *in vitro* leaf extract-treated AGS cells using an Annexin V-fluorescein isothiocyanate (FITC) apoptosis detection kit (BDPharmingen, CA, USA). Approximately 5×10^5^ cells were seeded into a 60-mL culture dish 24 h before being treated with extract. After 24 h, cells were trypsinized, washed with cold PBS and resuspended in 100 μL binding buffer. The cells were then stained with Annexin V-FITC and PI each in a Ca^2+^-enriched binding buffer. The cells were then incubated for 15 min in dark, 400 μL binding buffer was added and the assay was begun immediately using a FACScan flow cytometer (Becton Dickinson). The staining emissions were detected in the FL-1 and FL-3 channels. Approximately 10,000 counts were made for each sample. The distribution of live, early apoptosis, late apoptosis and necrotic cells were analysed.

### Withanomics database

The Withanomics database was created to serve as a public web resource for *Withania* transcriptome data. This database was designed using PHP (v4.3.9) and MySQL (v4.1.20). The front-end language PHP was connected with back-end MySQL by the Apache web server. The annotation, expression and other analysis data are stored in MySQL as tables, and this database is currently hosted on a CentOS (v5.8) Linux operating system. This database can be accessed at http://im-crop.snu.ac.kr/withania/index.php.

## Additional files

Additional file 1: Figure S1. Differentially expressed transcripts between *in vitro* root and leaf tissues. Additional file 2: Figure S2. The expression of the transcripts related HMGR, FPPS, SE, CAS, GT in *in vitro* root and leaf tissues are shown in heatmap. Additional file 3: Figure S3. HPTLC chromatogram of standards, withaferin A and withanolide A and 45-day-old *in vitro* leaf and root samples. A - Chromatogram of standard withaferin A; B - Chromatogram of standard withanolide A; C - Chromatogram of 45-day-old *in vitro* leaf sample indicating the presence of withaferin A; D - Chromatogram of 45-day-old *in vitro* root sample indicating the presence of withanolide A. Additional file 4: Table S1. Primers used for qRT-PCR analysis.

## Abbreviations

HMGR: Hydroxymethylglutaryl-CoA reductase
FPPS: Farnesyl-diphosphate synthase
SE: Squalene monooxygenase
CAS: Cycloartenol synthase
GT: Glucosyltransferases
GAPDH: Glyceraldehyde 3-phosphate dehydrogenase
SSR: Single sequence repeat
