# Supplementary figures and images for: Transcriptome analysis reveals in vitro cultured Withania somnifera leaf and root tissues as a promising source for targeted withanolide biosynthesis
Source: BMC Genomics. 2015 Jan 22;16(1):14. doi: 10.1186/s12864-015-1214-0 (PMC4310147; doi:10.1186/s12864-015-1214-0)

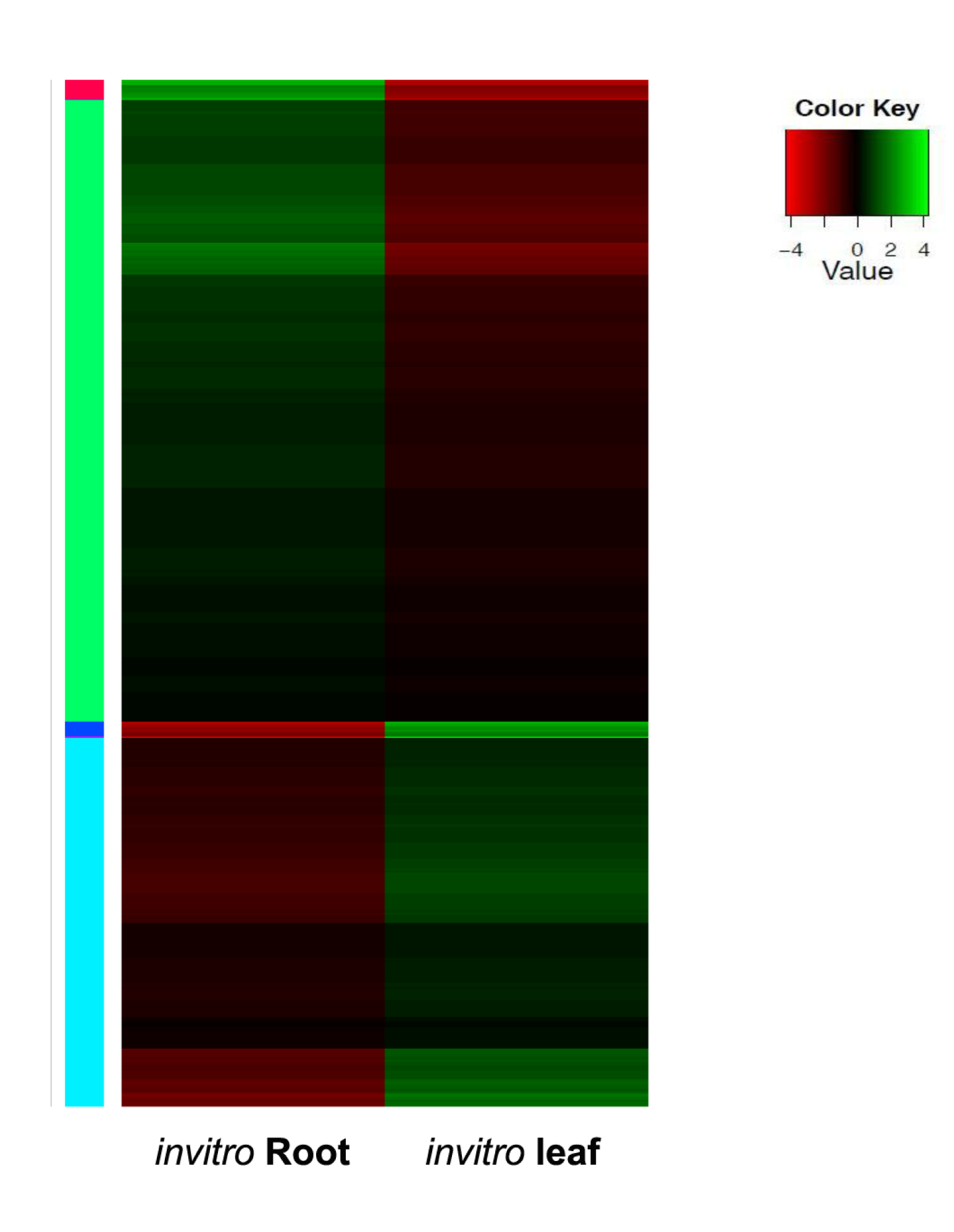

Supplement: Additional file 1: Figure S1. — Differentially expressed transcripts between in vitro root and leaf tissues. [file 12864_2015_1214_MOESM1_ESM.tiff]

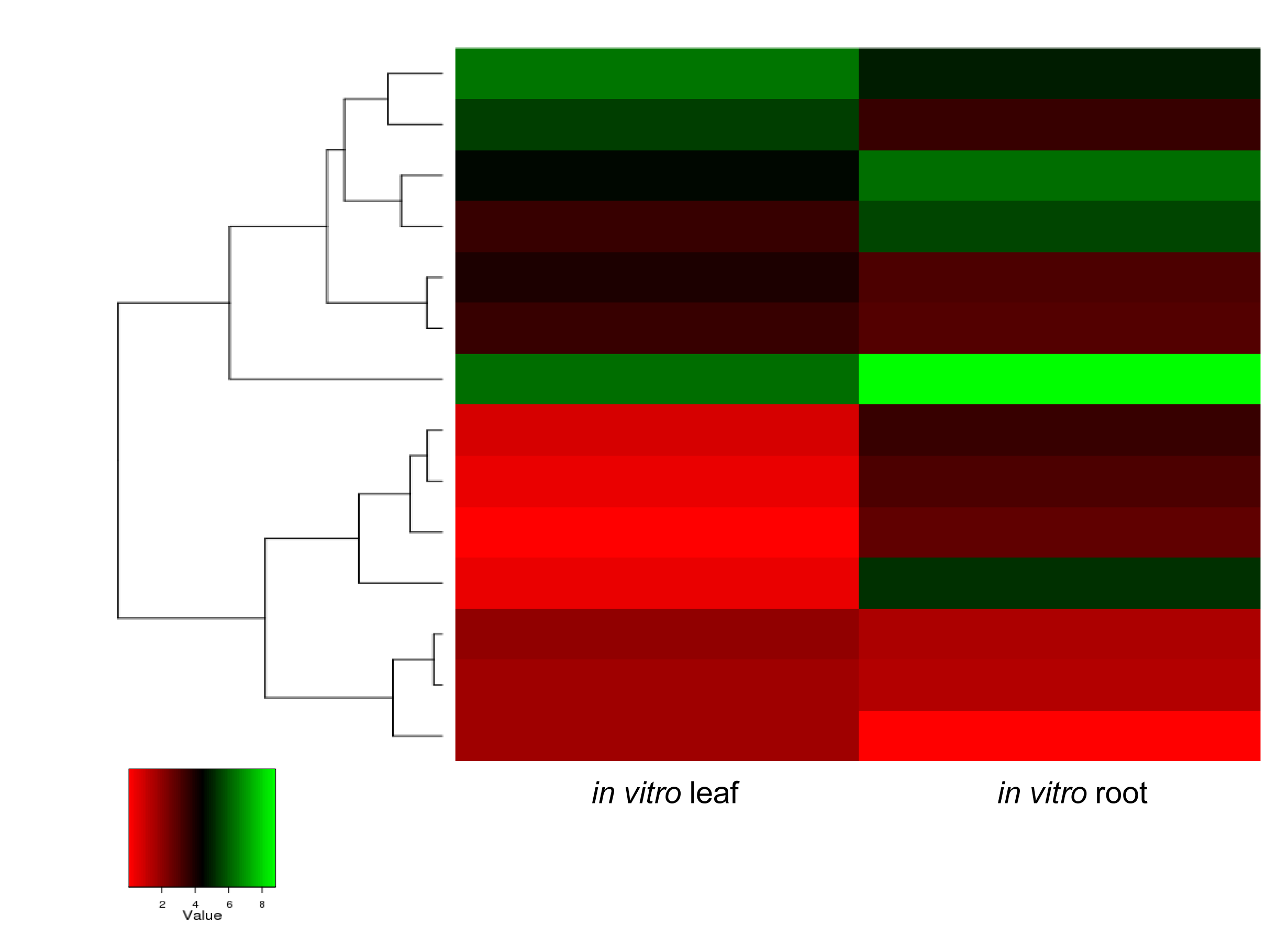

Supplement: Additional file 2: Figure S2. — The expression of the transcripts related HMGR, FPPS, SE, CAS, GT in in vitro root and leaf tissues are shown in heatmap. [file 12864_2015_1214_MOESM2_ESM.tiff]

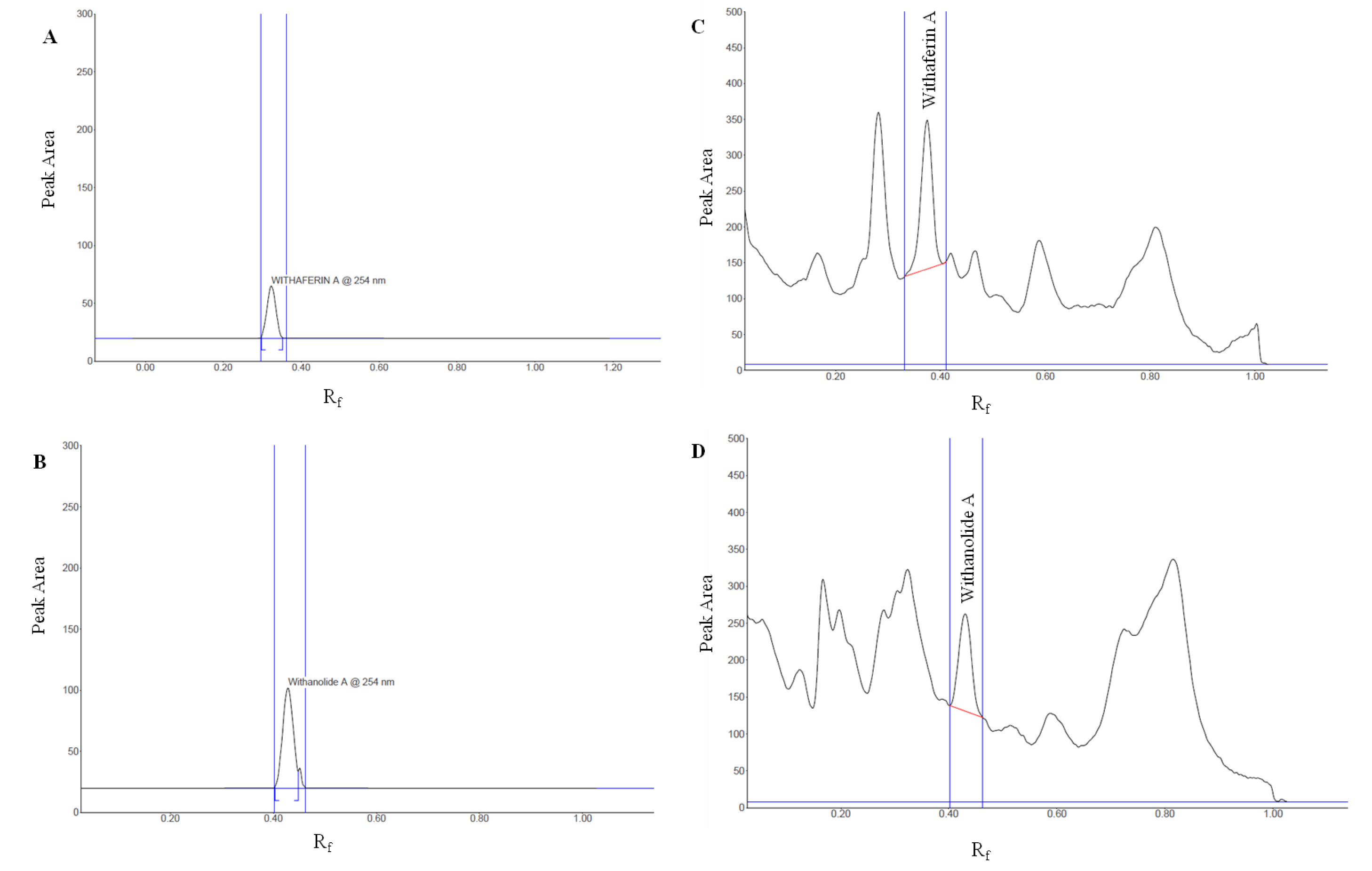

Supplement: Additional file 3: Figure S3. — HPTLC chromatogram of standards, withaferin A and withanolide A and 45-day-old in vitro leaf and root samples. A - Chromatogram of standard withaferin A; B - Chromatogram of standard withanolide A; C - Chromatogram of 45-day-old in vitro leaf sample indicating the presence of withaferin A; D - Chromatogram of 45-day-old in vitro root sample indicating the presence of withanolide A. [file 12864_2015_1214_MOESM3_ESM.tiff]
