# Supplementary material for: Transcriptome analysis reveals in vitro cultured Withania somnifera leaf and root tissues as a promising source for targeted withanolide biosynthesis
Source: BMC Genomics. 2015 Jan 22;16(1):14. doi: 10.1186/s12864-015-1214-0 (PMC4310147; doi:10.1186/s12864-015-1214-0)
Supplement: Additional file 4: Table S1. — Primers used for qRT-PCR analysis. [file 12864_2015_1214_MOESM4_ESM.doc]

**Additional file 4: Table S1.** Primers used for qRT-PCR analysis.

| **Primer name** | **Primer sequence** | **Amplicon size** |
| --- | --- | --- |
| **Ws GT** | Forward primer-  5' GTTTTCCTTCTTGCCGAGTG 3'  Reverse Primer –  5' AGGTCCCAGTCCCTTTTCAT 3' | 183 |
| **Ws HMGR** | Forward primer –  5' TGCTGCCAATATCGTCTCTG 3‘  Reverse Primer –  5' CCGTCACTGATAGCCTCCAT 3' | 105 |
| **Ws FPPS** | Forward primer –  5'TCGGGGGCTATCTGTTATTG 3'  Reverse primer –  5'CTCGGACGTGTATGGGAGTT 3’ | 165 |
| **Ws SE** | Forward primer –  5’CTGGTCCTGGAGAACTGC -3’  Reverse primer–  5’GGCTGATCCATCACCAATCT-3’ | 146 |
| **Ws CAS** | Forward primer –  5'GCCTGGCTTGATTATTGCTC 3'  Reverse Primer –  5'CACCCACCATCACTGTTCTG 3' | 117 |
| **GAPDH** | Forward primer –  5'CTCCATCACAGCCACTCAGA 3'  Reverse Primer –  5'GGTAGCACTTTCCCAACAGC 3' | 129 |
